# Annual Meeting of the Erie County Medical Society

**Published:** 1868-12

**Authors:** 


ANNUAL MEETING OF THE ERIE COUNTY MEDICAL SOCIETY.—The Annual Meeting of the Erie County Medical Society will be held January 11th, 1869, at the rooms of the Buffalo Medical Association. All regular practitioners of medicine in Erie county should attend; and especially those who have never yet joined this Society should take this opportunity to do so. There are many physicians who hold diplomas from the various medical colleges, who have never yet completed membership with the County Society and who are thus technically outside the fellowship of the profession. We cannot too strongly urge all such to attend this meeting and comply with the simple requirements of the law.

